# Two-Layer Sustained-Release Microneedles Encapsulating Exenatide for Type 2 Diabetes Treatment

**DOI:** 10.3390/pharmaceutics14061255

**Published:** 2022-06-13

**Authors:** Han Liu, Suohui Zhang, Zequan Zhou, Mengzhen Xing, Yunhua Gao

**Affiliations:** 1 Key Laboratory of Photochemical Conversion and Optoelectronic Materials, Technical Institute of Physics and Chemistry of Chinese Academy of Sciences, Beijing 100190, China; liuhan182@mails.ucas.ac.cn (H.L.); suohuizhang@mail.ipc.ac.cn (S.Z.); zhouzequan16@mails.ucas.edu.cn (Z.Z.); mengzhen@mail.ipc.ac.cn (M.X.); 2 University of Chinese Academy of Sciences, Beijing 100049, China; 3 Beijing CAS Microneedle Technology Ltd., Beijing 102609, China

**Keywords:** type 2 diabetes, sustained-release, two-layer microneedles, sodium alginate, exenatide

## Abstract

Daily administration of multiple injections can cause inconvenience and reduce compliance in diabetic patients; thus, microneedle (MN) administration is favored due to its various advantages. Accordingly, the two-layer sustained-release MNs (TS-MNs) were fabricated by encapsulating exenatide (EXT) in calcium alginate (CA) gel in this work. The TS-MNs were composed of a sodium alginate (SA) tip and a water-soluble matrix-containing calcium chloride (CaCl_2_). Subsequently, the calcium ion (Ca^2+^) contained in the matrix layer penetrated the tip layer for cross-linking, leaving the drug in the cross-linked network. The patches have adequate mechanical strength to pierce the skin; then, the matrix layer is dissolved, leaving the tip layer to achieve sustained release. Additionally, the TS-MNs encapsulating EXT retained high activity during long-term storage at room temperature. The pharmacokinetic results indicated that the plasma concentrations of EXT were sustained for 48 h in the EXT MN group, which agreed with the in vitro release test. Furthermore, they had high relative bioavailability (83.04%). Moreover, the hypoglycemic effect was observed to last for approximately 24 h after a single administration and remained effective after multiple administrations without drug resistance. These results suggest that the TS-MNs are a promising depot for the sustained delivery of encapsulated EXT.

## 1. Introduction

Diabetes, a metabolic disease characterized by hyperglycemia, will affect 700 million people in 2045 [1]. Typically, patients with type 2 diabetes account for more than 90% of people with diabetes. Poor glycemic control brings about severe complications, causing enormous risk and high mortality [2]. The glucagon-like peptide (GLP-1), as a new target for blood glucose regulation, has attracted increasing attention, and related drugs are also emerging [3]. Exenatide is an effective GLP-1 receptor agonist with 39 amino acids and 53% homology with humans that is currently approved by the FDA and the European Medicines Agency for glycemic control [4]. The product is administered by subcutaneous injection twice daily, and frequent administration for a long time may cause poor patient compliance [5]. Song reported that an oral CSK-DEX-PLGA nanoparticle carrier loaded with EXT was developed. However, the relative bioavailability of EXT in this nanoparticle carrier was only 9.2% compared with subcutaneous injection due to the gastrointestinal first-pass effect [6]. Although oral administration can notably enhance patient compliance, poor enzyme stability and gastrointestinal barriers result in relatively low availability [7,8]. Accordingly, a transdermal delivery system that can reduce patient injection pain, be administered autonomously, and maintain drug release and correspondingly improve bioavailability should be developed [9].

As micron-sized structures, unlike other transdermal delivery methods, MNs can break through the skin barrier and only pierce the epidermis in a minimally invasive manner without damaging neurons in the dermis, thereby minimizing pain associated with transdermal delivery, showing improved skin permeability and enhancing transdermal delivery [10,11,12]. Accordingly, MNs, especially biodegradable polymer MNs, have been extensively applied and are promising for the delivery of several macromolecules such as vaccines, peptides, and genes [13,14,15]. To realize the sustained release of drugs, various biodegradable materials were made into separable two-layer MNs composed of a slow-release tip and soluble substrate. Li reported that the separable MNs were composed of PLGA/PLA as a sustained-release tip material encapsulated levonorgestrel and soluble PVP/sucrose as a matrix material to realize the drug’s slow release [16]. Chen developed a separable MN composed of chitosan as the tip material to wrap ovalbumin and soluble PVA/PVP as the upper matrix material to realize drug delivery [17]. For small doses, the preparation of two-layer MNs can concentrate drugs at the MN tips to increase drug utilization.

Alginate is a naturally occurring high-molecular polysaccharide polymer that is ordinarily obtained from the cell walls of brown seaweed. The polymer has several unique advantages, including low toxicity, correspondingly low cost, biocompatibility, nonimmunogenicity, and biodegradation that have let it be extensively investigated and used for numerous biomedical and pharmaceutical applications [18,19]. Additionally, there are abundant studies on alginate as a sustained-release carrier, mostly including gel microspheres [20], nanoparticles [21], nanoaggregates [22], and microcapsules [23], etc. These carriers enter the body mainly by injection or oral administration. Moreover, as an anion polymer, alginate can be a hydrogel under mild conditions by adding divalent cations. This approach contributes to maintaining the activity of the biomolecules (e.g., enzymes, proteins, and peptides) encapsulated in the alginate gel [20]. However, few cross-linked alginate MNs have been studied. Zhou prepared gel-encapsulating coated MNs by coating with the water-soluble drug in an SA in situ complexation gel to achieve sustained release [24]. Due to these obvious properties, alginate has excellent potential value in drug sustained-release applications.

In this study, the TS-MNs were first fabricated by using SA as the MN tip material and soluble PVA/PVP/CaCl_2_ as the matrix material. Gelation of SA occurs rapidly in the presence of Ca^2+^; therefore, the in situ gel can hardly be poured into the mold to produce MNs due to the lack of fluidity [25]. Accordingly, the selected tip solution of SA was previously injected into the PDMS mold. Subsequently, the matrix solution containing Ca^2+^ was poured after removing excess solution. The Ca^2+^ contained in the matrix layer penetrated the tip layer for cross-linking, leaving the drug in the cross-linked network to achieve the sustained-release effect. After insertion, cross-linked MN tips were embedded in the skin after the matrix layer dissolved, followed by slow drug release [26]. Simultaneously, the TS-MNs encapsulating EXT also were first proposed, offering an effective alternative to regulate blood glucose. Herein, the effects of matrix solution concentration and CaCl_2_ concentration on gel content and drug diffusion and SA concentration on tip height and implantation efficiency were studied. Moreover, the insertion capability, in vitro release ability, activity, and stability of TS-MNs were investigated, and the activity in vivo was also evaluated.

## 2. Materials and Methods

### 2.1. Materials and Animals

EXT was obtained from Changchun Baiyi Pharmaceutical Co., Ltd. (Changchun, China). Sodium alginate (SA, 109 mPa·s) was acquired from Huanghai Biopharmaceutical Co., Ltd. (Qingdao, China). Polyvinyl alcohol (PVA, 6 mPa·s) was obtained from Jiangxi Alpha Hi-Tech Pharmaceutical Co., Ltd. (Pingxiang, China). Polyvinylpyrrolidone (PVP K90, Mv approximately 1000 kDa) was acquired from Boai NKY Pharmaceuticals Co., Ltd. (Jiaozuo, China). Polydimethylsiloxane (PDMS) was obtained from Dow Corning (Midland, TX, USA). Rhodamine B and calcium chloride (CaCl_2_) were purchased from Yinghai Fine Chemical Factory (Beijing, China). Fluorescein isothiocyanate (FITC) was bought from Beijing InnoChem Science & Technology Co., Ltd. (Beijing, China). Acetonitrile (HPLC grade) was purchased from Thermo Fisher Scientific (Waltham, MA, USA). STZ was acquired from Coolaber Technology Co., Ltd. (Beijing, China). Exendin-4 enzyme immunoassay (ELISA) kits were obtained from Phoenix Pharmaceuticals, Inc. (EK-070-94, Burlingame, CA, USA). Porcine cadaver skin was purchased from Kaikai Science and Technology Trading (Shanghai, China).

Sprague Dawley (SD) male rats (7 weeks old, approximately 200 g) were supplied by the SPF Biotechnology Co., Ltd. (Beijing, China). All rats were housed under specific pathogen-free conditions with free access to food and water. The Institutional Animal Care and Utilisation Committee of the Technical Institute of Physics and Chemistry, CAS (approval number, IACUC-IPC-21042; 16 May 2021, and IACUC-IPC-21061; 27 June 2021) approved the study protocol.

### 2.2. Fabrication of TS-MNs

**Figure 1:**
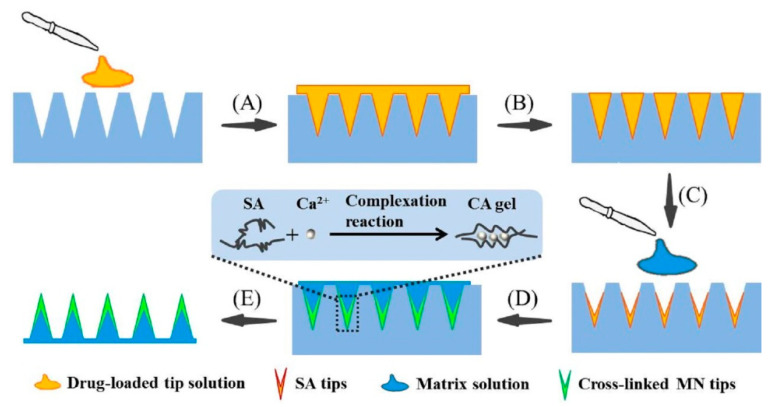
Fabrication process schematic of TS-MNs. (**A**) The drug-loaded tip solution was dropped onto the mold surface under vacuum conditions. (**B**) The superfluous tip solution was scraped off. (**C**) The mold was dried at room temperature with approximately 20% humidity for 30 min. (**D**) The matrix solution was cast in the mold and the CA gels were formed by the complexation reaction of SA and Ca^2+^. (**E**) The TS-MNs were stripped from the mold.

The novel TS-MNs were composed of SA tip material encapsulating EXT and soluble PVA/PVP/CaCl_2_ matrix material. The tip material was obtained by dissolving 3% (wt) SA in an aqueous solution with an EXT content of 2% (wt). To attain the matrix material, the excipients consisting of 17.5% (wt) PVA, 17.5% (wt) PVP, and 4% (wt) CaCl_2_ were dissolved in deionized water. The MN fabrication process is depicted in Figure 1A–E. The TS-MNs were created by initially dropping 10 μL drug-loaded tip solution onto the PDMS mold, and mold cavities were filled with the tip solution under vacuum conditions for 10 min. Then, the superfluous tip solution remaining on the mold surface was scraped off to make the first layer. Subsequently, the mold was placed at room temperature with approximately 20% humidity for drying for 30 min. Finally, 40 μL of the prepared matrix solution was cast in the mold under vacuum conditions for 10 min to form the second layer. After drying at room temperature, the TS-MNs were acquired by stripping from the mold.

### 2.3. Effect of Matrix Solution Concentration on Gel Content

A series of 25%, 30%, and 35% (wt) matrix solution was obtained by dissolving PVA and PVP (mass ratio 1:1) in a specific mass of deionized water. Subsequently, 4% (wt) CaCl_2_ powder was added to the above matrix solution. One milliliter of the acquired matrix solution (PVA/PVP/CaCl_2_) was put into EP tubes. The sodium alginate MNs (SA-MNs) were fabricated based on Figure 1A. Briefly, 30 μL SA solution (3%) was dropped onto the PDMS mold. After drying, SA-MNs were removed from the mold and used. The acquired SA-MNs were placed in open empty EP tubes and dried in an oven at 40 °C overnight; then, these patches were weighed (W_0_). In addition, SA-MNs were placed vertically in EP tubes containing 1 mL matrix solution, with the tip facing down (Figure S1). Subsequently, cross-linked SA-MNs were removed from the tubes at specific time points of 10, 30, 60, 120, 240, and 360 min, and the excess solution was wiped off the surface. The wiped patches were washed with water four times by 1500 rpm vortex for 10 min each the previous two times and 2 h each the final two times. The washed patches were similarly placed in an oven at 40 °C for drying overnight and were weighed (W_e_). The gel content was achieved by using Equation (1) [27].

(1) $${\mathrm{Gel}\;\mathrm{content}\;(\%)=W}_{e}/W_{0}\times 100\%$$

### 2.4. Effect of Matrix Solution Concentration on Drug Diffusion

To observe the diffusion intuitively of drugs in MNs in matrix solution with different concentrations, rhodamine B with a distinct color was selected as the model drug [28]. The casting material was obtained by mixing 3% (wt) SA solution with rhodamine B (2 mg/mL). SA-MNs containing rhodamine B were prepared based on the methods described in Section 2.3. The patches containing rhodamine B were placed in the EP tube of the matrix solution containing Ca^2+^, and the dye diffusion state was observed visually after 0 h and 10 h. To make the diffusion phenomenon more practical, the TS-MNs encapsulating rhodamine B were applied, which was attained based on the preparation method in Section 2.2.

### 2.5. Effect of CaCl_2_ Concentration on Gel Content

Matrix material containing different CaCl_2_ concentrations was obtained by adding 1%, 2%, 3%, and 4% (wt) CaCl_2_ powders to PVA/PVP matrix solution with a concentration of 35% (wt). The TS-MNs were acquired according to the preparation method described in Section 2.2. As the tips were difficult to collect and analyze, the excess solution was not removed after dropping the 30 uL tip solution described in this section. The acquired MNs were washed with water five times by 1500 rpm vortex for 5 min each time the first three times and 2 h each time the last two times. The washed patches were placed in an oven at 40 °C for drying overnight and were weighed (W_e1_). The gel content was similarly acquired from Equation (1).

### 2.6. Influence of SA Concentration on Tip Height and Implantation Efficiency

The TS-MNs were prepared in the same way as described in Section 2.2, where the SA concentration was 2%, 3%, 4%, and 5% (wt), respectively. The TS-MNs with different prescriptions were dissolved in water and swirled for 5 min; then, the length of partial tips was measured under the fluorescence microscope to acquire the height of the cross-linked tip. The implantation efficiency of TS-MNs was evaluated by rat skin in vivo (*n* = 4). The total number of tips (Q_t_) was recorded and the TS-MNs were inserted into rat abdominal skin using a homemade applicator (20 N/cm^2^) for 30 s. The rat abdomen was wrapped with medical tape. After staying for 30 min, the TS-MNs were removed from the rat and observed under a microscope, and the number of tips (Q_r_) that were not implanted into the skin was recorded. The implantation efficiency was calculated based on Equation (2).

(2) $$\mathrm{Implantation}\;\mathrm{efficiency}\;\left(\%\right)=(Q_{t}-Q_{r})/Q_{t}\times 100\%$$

### 2.7. Morphology Analysis and Skin Insertion Tests

#### 2.7.1. Preparation of FITC-EXT

First, 0.15 M NaCl solution was obtained by dissolving 0.44 g NaCl in 50 mL water; 0.15 M NaHCO_3_-Na_2_CO_3_ solution was acquired by dissolving 0.80 g Na_2_CO_3_ in 5 mL water and 0.63 g NaHCO_3_ in 45 mL water, and they were mixed and adjusted to pH = 9.0; 10 mg/mL EXT solution was obtained by dissolving 0.03 g EXT in 3 mL (V_0.15 M NaCl_:V_0.15 M NaHCO3-Na2CO3_ = 9:1) solution. Then, 0.4 mg FITC was placed in a glass bottle containing 3 mL EXT solution and reacted with magnetic stirring at 4 °C for 12 h away from light. The reacted FITC-EXT solution was placed in a dialysis bag, and dialyzed at 4 °C away from light. Water was changed twice a day until no fluorescence was detected. The acquired FITC-EXT solution was filtered and entered the HPLC system for quantification. The experimental conditions for HPLC were similarly depicted in Section 2.8.

#### 2.7.2. Morphology Analysis and Skin Insertion Tests

The structural shape of TS-MNs was analyzed using a stereomicroscope (SMP1000, Nikon, Tokyo, Japan) and a fluorescence microscope (BX51, Olympus, Tokyo, Japan). To assess the skin insertion ability, TS-MNs loaded with FITC-EXT were inserted into porcine skin ex vivo (800 μm thick) using a homemade applicator (20 N/cm^2^) for 30 s. The skin after treatment of MNs was placed on an agarose hydrogel (2% wt) to keep it moist for 30 min; then, the residual MNs were removed from the porcine skin. The porcine skin surface and the residual MNs were photographed with a fluorescence microscope.

The mechanical performance of TS-MNs was assessed using a displacement–force test station (1220SB, Nantian Testing Instrument Co., Ltd., Dongguan, China). The procedures and conditions of the experiment were detailed in previous studies [29].

To prepare histological sections, the porcine skin area treated with TS-MNs containing FITC-EXT was cut off, embedded with OCT complex at −80 °C for several minutes, and then placed in a cryostat mold for 20 min. The frozen OCT-skin was cut into 15 μm slices by a freezing microtome (CM3050S, Leica, Brunswick, Germany) and placed on a cover glass. The samples were viewed using a fluorescence microscope.

### 2.8. Quantification of EXT by HPLC

The contents of EXT encapsulated in the TS-MNs were released by dispersing one patch in 1 mL PBS solution and vortex for 8 h. Then, the solution was filtered and entered the HPLC (LC-20AT, Shimadzu, Tokyo, Japan) system for analysis. A YMC Triart C18 (250 mm × 4.6 mm, 5 μm) column was applied to separate EXT at 40 °C. Chromatographic separation was adopted by the mobile phases (A) 0.05 M KH_2_PO_4_ solution (pH = 4.5) and (B) acetonitrile at the following gradient elution: 1–12 min, B: 35–50%; 12–17 min, B: 50%; 17–18 min, B: 50–35%; and 18–25 min, B: 35%. The injection volume was 20 μL, the flow rate was 1 mL/min, and the wavelength was detected at 218 nm.

### 2.9. In Vitro Release and Kinetics Study

The release of EXT encapsulated in MNs was carried out using the dialysis bag method. Specifically, the tips of TS-MNs were scraped off with a scalpel and placed in the dialysis bag; then, the dialysis bag was hung in a supported conical flask containing 2 mL PBS (pH = 7.4) receiving the solution during incubation at 37 °C with gentle magnetic stirring [30]. The entire solution was collected from the conical flask at the specified time point; subsequently, the same amount of PBS was added to the conical flask. The acquired solution was filtered and entered the HPLC system for EXT quantification. The experimental conditions for HPLC were similarly depicted in Section 2.8.

To study the kinetics and mechanism of EXT release from the TS-MNs, the in vitro release data were fitted into three common mathematical models [31,32]. The kinetic models include zero-order kinetics (Equation (3)), first-order kinetics (Equation (4)), and Higuchi kinetics (Equation (5)).

Q_t_ = kt (3)

Q_t_ = n [1 − e^(−kt)^] (4)

Q_t_ = kt^1/2^ + A (5)

where Q_t_ is the cumulative release rate of the drug within time t; k is the release rate constant; n is a constant; A is a simplified constant.

### 2.10. Determination of EXT Activity and MN Storage Stability

The secondary structure of EXT was determined by a CD spectrophotometer (Jasco J-815 Spectrophotometer, Tokyo, Japan). The method for extracting EXT from TS-MNs was similarly described in Section 2.8. The unprocessed EXT solution (concentration of 200 μg/mL) was directly prepared with PBS buffer (pH = 7.4) as an EXT activity control. The EXT solution was injected into a quartz sample cell with a slit of 1 mm and scanned by CD at room temperature. The instrument parameters were set as follows: bandwidth of 1 nm, scanning speed of 100 nm/min, scanning times of 3 times, and detection wavelength of 195–260 nm. The blank PBS solution was used to correct the baseline. To evaluate the storage stability of TS-MNs containing EXT, MNs were first heat-sealed and packaged and then kept at 4, 25, and 40 °C for one and two months, respectively. Then, EXT was extracted from the MNs and detected as described in Section 2.8.

### 2.11. In Vivo Pharmacokinetics

The rats received adaptive feeding for one week after arrival, and they were anesthetized with ether. Their abdominal hair was removed and treated with depilatory cream. During the experiment, the animals were given optional food and water. The rats were randomly divided into two groups (*n* = 6): (1) the subcutaneous (SC) injection group and (2) the EXT MN group. Each animal in the SC injection group was given 10.00 μg/0.5 mL EXT solutions subcutaneously using a 1 mL syringe. In the EXT MN group, two patches (total 38.96 μg) were inserted into the skin of each rat using a homemade applicator (20 N/cm^2^) for 30 s. The MNs were removed from the rat after 30 min. Blood samples were collected from the caudal vein at time points (0.5, 1, 2, 4, 6, 8, 12, 16, 24, 36, and 48 h after dosing). They were collected in a heparin sodium tube, centrifuged at 3000 rpm for 10 min, and then stored at −20 °C before analysis. The plasma EXT level was quantified using an ELISA kit according to the instructions. The relative bioavailability (RB) was calculated by Equation (6):

(6) $$\mathrm{RB}=({\mathrm{AUC}}_{\mathrm{MN}}\times {\mathrm{Dose}}_{\mathrm{SC}})/({\mathrm{AUC}}_{\mathrm{SC}}\times {\mathrm{Dose}}_{\mathrm{MN}})\times 100\%$$

### 2.12. In Vivo Pharmacodynamics

#### 2.12.1. Establishment of the Type 2 Diabetes Rat Model

After four weeks of high-fat diets, all the rats were intraperitoneally injected with 25 mg/kg STZ solution within half an hour. The STZ solution was obtained by dissolving STZ in citrate buffer until it was ready for use. After 72 h, the same dose of STZ solution was injected again. One week later, glucose levels were detected by a Yuwell glucose meter (Jiangsu, China), and fasting blood glucose ≥ 16.7 mmol/L was considered indicative of diabetes [33].

#### 2.12.2. Single Administration in Diabetic Rats

To evaluate the efficacy of a single administration in diabetic rats, nonfasting diabetic rats were randomly divided into three groups (*n* = 6): the EXT MN group, the blank MN group, and SC injection group. After a detachment of the abdominal hair, the rats in the three groups were treated with EXT MNs (two patches for each, total 38.96 μg), blank MNs (two patches for each), or SC injections (10.00 µg/0.5 mL EXT solution for each), respectively. The MNs were removed from the rat after 30 min. Blood was collected at 0, 0.5, 1, 2, 3, 4, 6, 8, 12, 20, 24, 30, and 36 h after administration, and the blood glucose level was detected by a glucose meter. A normal diet and water were given after blood glucose was measured at 12 h.

#### 2.12.3. Multiple Administrations in Diabetic Rats

Ultimately, the therapeutic efficacy of MNs for multiple administrations was assessed. The overnight fasting diabetic rats were randomly grouped (*n* = 4) with EXT MN, blank MN, and SC injection. All animals in the three groups received short-term treatment for 6 days and were administered the same dose as a single administration at 9:30 a.m. daily. During the period after daytime administration, the rats were fed a free diet. The blood glucose level was measured by a biochemical analyzer at 0, 4, and 12 h after administration, and overnight fasting for 12 h was continued after the last glucose measurement daily.

### 2.13. Statistical Analysis

All the experimental values are presented as the mean ± SD. Microsoft Excel 2010 software was used to analyze statistical data. Origin 2018 software was used to plot graphs. A value of *p* < 0.05 was considered statistically significant.

## 3. Results and Discussion

### 3.1. Factors Affecting Gel Content and Drug Diffusion

**Figure 2:**
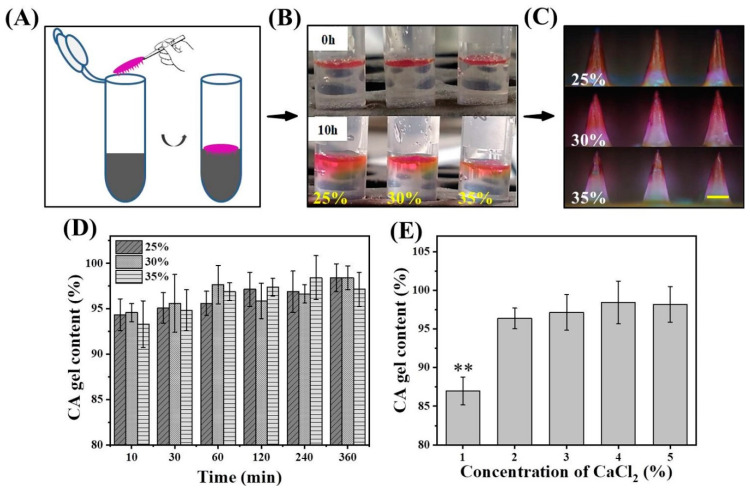
Factors affecting CA gel content and drug diffusion. (**A**) The SA-MN patch was placed in PVA/PVP/CaCl_2_ matrix solution, and a cross-linking reaction occurred. (**B**) Diffusion of rhodamine B loaded on SA-MNs in 25%, 30%, and 35% matrix solution at 0 h and 10 h. (**C**) Optical microscope image of TS-MNs consisting of SA tip material encapsulating rhodamine B (2 mg/mL) and soluble matrix material with different concentrations. Scale bars, 200 μm. (**D**) Influence of cross-linking time and matrix concentration on the CA gel content. (**E**) Influence of different CaCl_2_ concentrations in the matrix solution on the CA gel content. Each bar represents the mean ± SD (*n* = 4), ** *p* < 0.01.

The effects of PVP/PVA (mass ratio 1:1) concentration on the gel content and the diffusion of rhodamine B were first investigated under the condition of constant CaCl_2_ concentration. The experiment is to place the SA-MNs in PVA/PVP matrix solution containing calcium (Figure 2A), and the MNs can be removed at different time points after cross-linking. Thus, the CA gel content can be calculated and the diffusion of the model drug with color can be directly observed. In the preparation of two layers MNs, the drugs in the tip layer diffuse upward after adding the second layer of matrix solution, resulting in drug waste [34]. For a more intuitive observation of drug diffusion, the diffusion phenomenon of rhodamine B loaded on SA-MNs was recorded at 0 h and 10 h in 25%, 30%, and 35% mixed matrix solution (Figure 2B). The results showed that different concentrations of matrix solution impacted the drug diffusion. At 10 h, the diffusion rate of rhodamine B in matrix solution was in the order of 25% > 30% > 35% from fast to slow. Therefore, a higher concentration of matrix solution was arduous for the drug in the tip to penetrate it. As shown in Figure 2C, the color was inclined to be gathered in the tips as the content of the PVA/PVP solution increased from 25% to 30% and 35%. This indicated that the matrix material concentration affected the drug diffusion partly, which agreed with the results of Figure 2B. Additionally, the matrix solution with a high concentration could effectively inhibit drug diffusion and increase the drug utilization rate [28].

The influence of the matrix solution with different concentrations on CA gel content at different times is shown in Figure 2D. Because of the rapid complexation between alginate and Ca^2+^, more than 90% CA gel content was formed in the 25%, 30%, and 35% matrix solution after 10 min. Before 60 min, the CA gel content increased with the extension of reaction time, although the increment was small, and there was no significant change in the subsequent trend. Besides, different matrix solution concentrations had no significant effect on CA gel content, probably due to the fast cross-linking process; thus, the matrix solution concentration did not affect the CA gel content. Consequently, the cross-linking rate was not affected by the concentration of the matrix solution, but drug diffusion was affected. Accordingly, PVA/PVP (mass ratio 1:1) matrix solution with a concentration of 35% was selected as the second layer to prepare TS-MNs.

The influence of calcium concentration in the upper matrix solution on CA gel content was further discussed when other conditions were consistent. The calcium concentration affected the CA gel content, and the content could be calculated by cross-linking with SA by adding different concentrations of CaCl_2_ to the 35% mixed matrix solution. According to Figure 2E, the CA gel content formed was 86.98 ± 1.80% when the CaCl_2_ concentration was 1%. When the CaCl_2_ concentration exceeded 2%, the content increased slightly, reaching above 96% in all instances. When the CaCl_2_ concentration increased to 4%, the content reached 98.44 ± 2.76%. Then, with increasing CaCl_2_ concentration, the CA gel content remained unchanged.

### 3.2. Influence of SA Concentration on Tip Height and Implantation Efficiency

**Figure 3:**
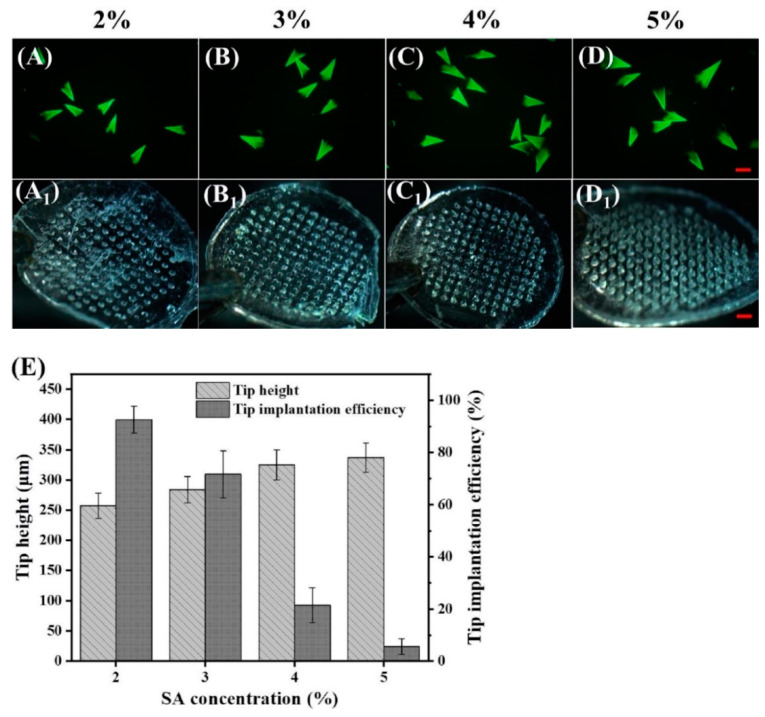
Influence of SA concentration on tip height and implantation efficiency. (**A**–**D**) Fluorescence morphology of tips in water with SA concentrations of 2%, 3%, 4%, and 5% respectively. The tip height could be measured directly by fluorescence microscopy. Scale bars, 200 μm. (**A_1_**–**D_1_**) Stereomicrograph of TS-MNs with different prescriptions acting on the skin (30 min after insertion). The number of tips that were not implanted into the skin could be counted from the stereomicrograph. Scale bars, 500 μm. (**E**) Tip height and implantation efficiency of TS-MNs. Each point represents the mean ± SD (*n* = 4).

The effect of SA concentration on the relationship between tip height and implantation efficiency was analyzed. After the TS-MNs dissolved in water, the water-soluble matrix material was dissolved, and the cross-linked CA tip remained. Accordingly, the tip height of MNs could be measured directly by fluorescence microscopy (Figure 3A–D). Thirty minutes after implantation, the number of tips that were not implanted into the skin after MN removal was observed under a microscope to calculate the implantation efficiency (Figure 3(A_1_–D_1_)). As shown in Figure 3E, the height of the cross-linked tips increased with increasing SA concentration, whereas the implantation efficiency decreased, which was an inverse relationship. The tip height and implantation efficiency were 257.56 ± 20.89 μm; 96.54 ± 1.86% and 337.08 ± 24.03 μm; and 5.68 ± 2.99%, which corresponded with SA concentrations of 2% and 5%, respectively. The difference was approximately 80 μm and 90%. It may be that the viscosity increased accordingly with the increase in SA concentration, resulting in more solution sticking to the wall on the PDMS mold after casting. Thus, the tip height improved after the cross-linking reaction with Ca^2+^. To simplify the delivery method and allow rapid MN detachment, the time required for the patch to stick to the skin needs to be reduced [35]. Consequently, considering the short-term implantation efficiency, a high-concentration SA solution is not recommended as the tip solution.

### 3.3. Characterization and Insertion Capability of TS-MNs

**Figure 4:**
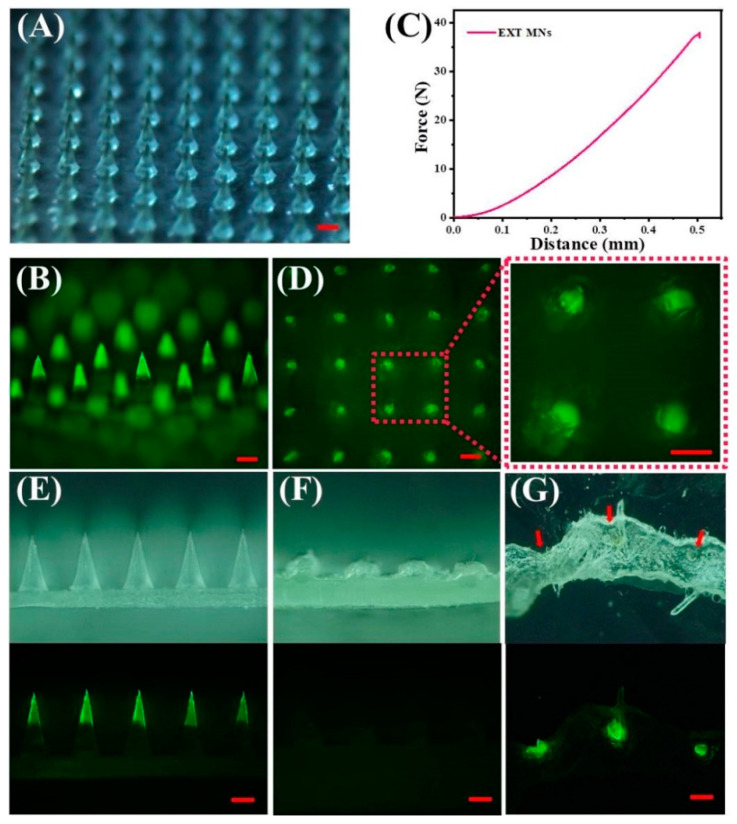
Characterization and insertion capability of TS-MNs. (**A**) Stereomicroscopic images of TS-MNs containing FITC-EXT. Scale bars, 200 μm. (**B**) Fluorescence images of TS-MNs encapsulated FITC-EXT. Scale bars, 200 μm. (**C**) Mechanical behavior of the TS-MNs. (**D**) Fluorescence images of porcine skin after TS-MNs application and removal. Scale bars, 200 μm. Side view of stereomicroscope (above) and fluorescence microscopy (below) of TS-MNs before (**E**) and after (**F**) skin insertion for 30 min. Scale bars, 200 μm. (**G**) Histological section of isolated porcine skin imaged by stereomicroscope (above) and fluorescence microscopy (below). Scale bars, 200 μm.

To evaluate the morphology and skin-piercing capability of the TS-MNs, FITC, a fluorescent dye, was grafted onto EXT and then encapsulated into the TS-MNs. After grafting, the concentration of FITC-EXT solution was 3.64 mg/mL. The TS-MNs loaded with FITC-EXT are shown in Figure 4A, with a substrate width of 300 μm, height of 600 μm, and tip spacing of 500 μm. Figure 4B shows that there is a boundary between the tip layer and the matrix layer, demonstrating that the FITC-labeled EXT was gathered in the MN tips. To ensure that the TS-MNs could successfully pierce the stratum corneum, the mechanical strength of the MNs was analyzed. As displayed in Figure 4C, the compression force increased to 37.90 N when displacement reached 500 μm, and the curve was not broken, indicating that the TS-MNs possessed sufficient strength to pierce the stratum corneum [36,37]. After the FITC-labeled EXT TS-MNs were implanted into the isolated porcine skin, a clear channel was left through the skin, showing that the tips had entered the skin (Figure 4D). Figure 4E,F show the side view of a stereomicroscope (above) and fluorescence microscope (below) before and after MN treatment on porcine skin, and no tips existed after MN treatment. Additionally, histological sections indicated that the TS-MNs entered the skin (Figure 4G). Together, these results suggest that the TS-MNs have adequate mechanical strength to pierce the skin.

### 3.4. In Vitro Release and Kinetics Study

**Figure 5:**
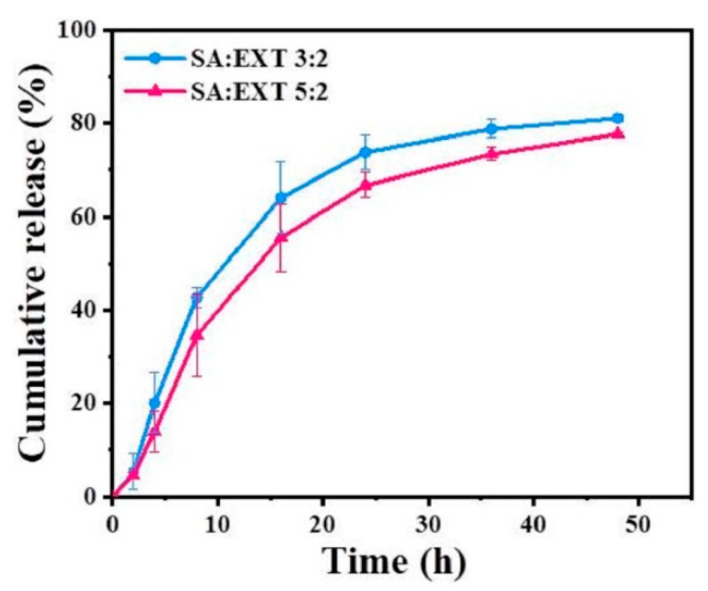
In vitro cumulative release profiles of EXT from TS-MNs with different SA-to-EXT ratios.

To investigate the release behavior of encapsulated EXT from TS-MNs, the in vitro release of MNs with various SA: EXT ratio was carried out. The polymer-to-drug ratio may affect the behavior of drug release [30]. As depicted in Figure 5, the release of EXT decreased as the SA: EXT ratio increased, indicating that the content of cross-linked gel formed increased with increasing SA content, and the drug was more difficult to diffuse. Consequently, the gel content affected the drug release certainly. In the first 24 h, the cumulative release of EXT with SA: EXT ratios of 3:2 and 5:2 were 73.80 ± 3.57% and 66.72 ± 2.69%, respectively. Subsequently, the release of EXT was slow, with cumulative releases of 81.06 ± 0.78% and 77.66 ± 0.48% at 48 h, respectively. After MNs were implanted into the skin, EXT at the MNs surface may be quickly absorbed by skin tissue fluid. Then, EXT in the MNs can gradually diffuse from the cross-linked CA gel [38].

The fitting results in Table 1 showed that the cumulative release curves of TS-MNs assisted EXT conformed to the first-order kinetics. This indicated that EXT was released at a slow and non-constant rate, in line with the drug release mode of sustained-release preparation.

### 3.5. Activity of EXT after MN Fabrication and Storage Stability of EXT Encapsulated in MNs

**Figure 6:**
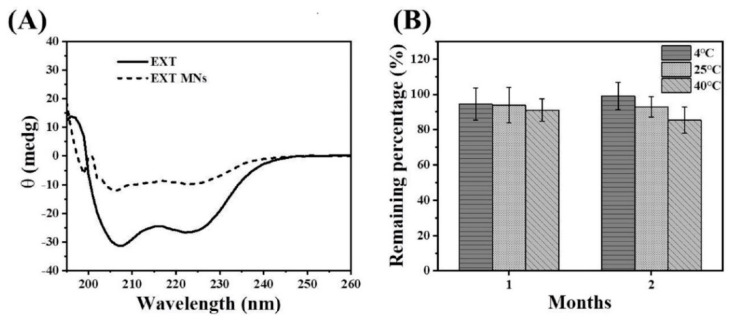
Assessment of EXT activity by CD and the storage stability of MNs via HPLC. (**A**) CD spectra of unprocessed EXT (concentration of 200 μg/mL) and EXT extracted from MNs (concentration of 50 μg/mL). (**B**) Storage stability of EXT-loaded MNs at 4, 25, and 40 °C for two months. Each point represents the mean ± SD (*n* = 4).

Peptide drugs are unstable, especially in the fabrication process, and experience a variety of instability factors, resulting in peptide aggregation, degradation, and inactivation [39]. To evaluate the secondary structure of EXT, circular dichroism (CD) was applied to examine the activity of EXT (Figure 6A). The results indicated that the retention time of the EXT extracted from the TS-MNs was the same as that of the unprocessed EXT. The maximum negative absorption peaks were approximately 208 nm and 224 nm, indicating that the secondary structure of the extracted EXT was not affected and that the activity of EXT was protected. The reason for the inconsistent retention time between the two near 200 nm is that a small amount of PVP material in the MN interferes with detection but does not affect the evaluation of the EXT secondary structure. To assess the long-term activity of EXT encapsulated in MNs, the TS-MNs were stored at different temperatures for two months, and the results are shown in Figure 6B. At one month post-storage, the remaining percentages of EXT were 94.42%, 93.81%, and 91.08% at 4, 25, and 40 °C, respectively. At two months, the activity of EXT was retained at 99.05%, 92.96%, and 85.34% at 4, 25, and 40 °C, respectively. After the TS-MNs were stored for one month, the remaining contents of EXT were maintained above 90%. Although the activity of EXT decreased slightly at 40 °C after two months, 85% of the activity remained.

### 3.6. In Vivo Pharmacokinetics

**Figure 7:**
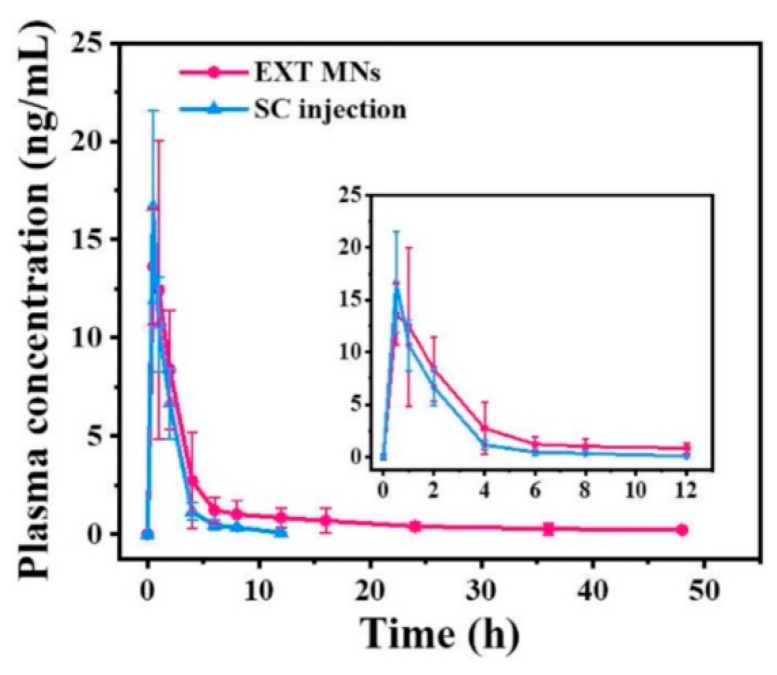
Plasma concentration of EXT versus time profiles in normal rats after treatment with SC injection (dosage: 10.00 µg) and EXT MNs (dosage: 38.96 µg) (*n* = 6).

The TS-MNs encapsulating EXT were embedded in normal mice to investigate EXT pharmacokinetics compared with SC injection. The pharmacokinetic curve and correlative pharmacokinetic parameters after administration are illustrated in Figure 7 and Table 2. The dose ingested in the EXT MN group (38.96 μg, the delivery efficiency of EXT was 60.89%, which was calculated by subtraction and not presented in detail in this work. Thus, the intake dose was 23.72 μg) was approximately twice that given in the SC injection group (10.00 μg). The maximum plasma concentration (C_max_) in the EXT MN treatment group and SC injection group was 13.60 ± 2.95 ng/mL and 16.68 ± 4.87 ng/mL, respectively. The peak levels (T_max_) were reached in both groups 0.5 h after administration. Additionally, the plasma concentration of EXT after treatment with MNs could be detected for 48 h, which agreed with the in vitro release test. With SC injection as a reference, the RB of TS-MNs encapsulating EXT was 83.04% based on calculated AUC values and intake doses, indicating that EXT released by the MNs entered the systemic circulation well.

### 3.7. Hypoglycemic Effect In Vivo

**Figure 8:**
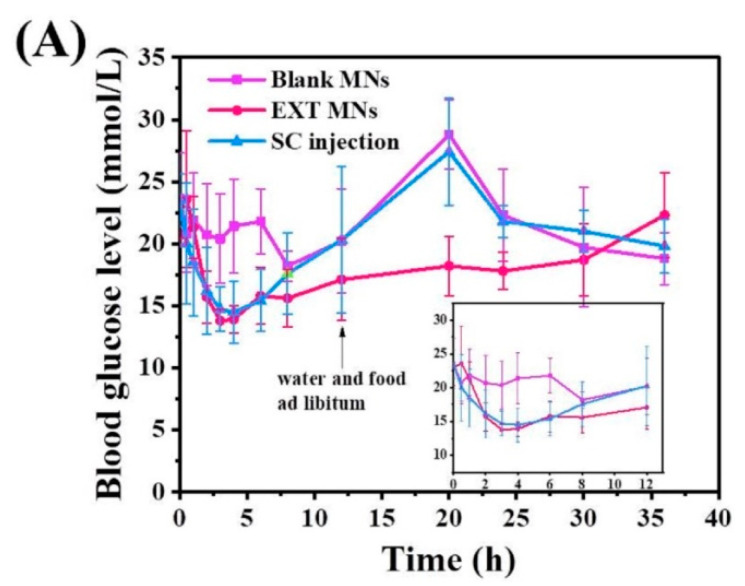

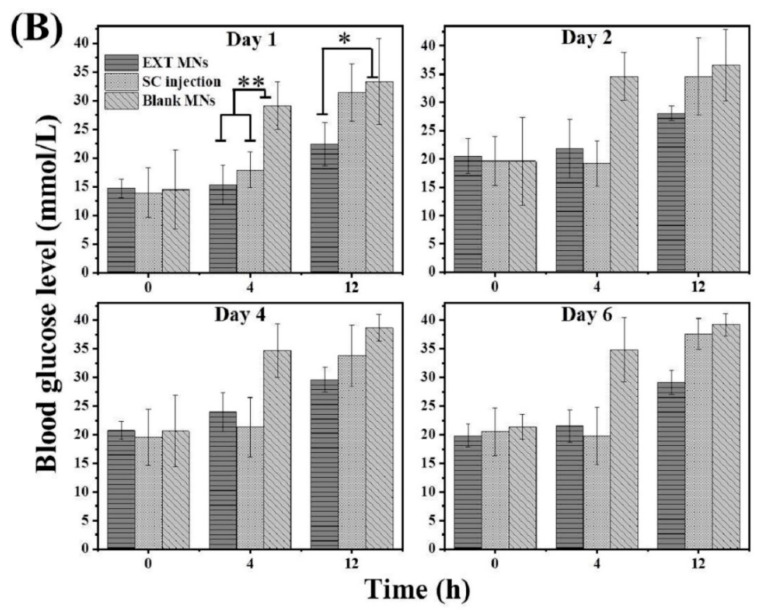
In vivo activity of EXT-loaded MNs. (**A**) Blood glucose levels of non-fasting diabetic rats after a single administration of SC injection, blank MNs, and EXT-loaded MNs (*n* = 6). (**B**) Short-term hypoglycemic efficacy of fasting diabetic rats (fasting from 9:30 p.m. to 9:30 a.m. every day) post-administration for six days (*n* = 4). Each bar represents the mean ± SD: * *p* < 0.05; ** *p* < 0.01.

The blood glucose levels versus time profiles after a single administration are displayed in Figure 8A. As expected, the blank control group showed no evident hypoglycemic effect before 12 h, and the blood glucose value measured the next day reached 28.8 ± 2.8 mmol/L at 20 h after food and water ad libitum, with typical hyperglycemia symptoms. After SC injection, blood glucose gradually decreased from 23.5 ± 2.1 mmol/L at 0 h to 14.5 ± 2.5 mmol/L at 4 h (approximately 38.19 ± 9.20%) and then recovered to a hyperglycemic state within 8 h. Similarly, the blood glucose value reached 27.4 ± 4.3 mmol/L on the second day post normal diet, indicating that the injection of EXT solution can maintain the blood glucose level for 8 h, probably due to the rapid clearance of drugs. In the group treated with EXT MNs, the blood glucose decreased from 23.0 ± 2.6 mmol/L at 0 h to 13.9 ± 1.1 mmol/L at 4 h (approximately 38.31 ± 10.71%). Due to the sustained release of the EXT MNs, the blood glucose level was still inhibited to 18.2 ± 2.4 mmol/L on the second day; the hypoglycemic effect lasted for approximately 24 h, after which the recovery was consistent with that of the blank control group.

To investigate the availability of EXT in regulating blood glucose in the short term, a daily administration was performed for six days. Blood glucose levels after multiple doses are shown in Figure 8B. Here, diabetic rats were rendered a free diet immediately after administration. At 4 h, the blood glucose level of the blank MN group increased significantly. However, that of the EXT MN group and the SC injection group was the same and showed no significant increase compared with the fasting blood glucose level, indicating effective control of postprandial blood glucose. With the continuous intake of food, the blood glucose levels in the blank MN and SC injection groups similarly continued to rise at 12 h, demonstrating that the SC injection group could not maintain the regulation of postprandial blood glucose. In the EXT MN group, the postprandial hypoglycemic effect remained as before, despite a slight increase in blood glucose. Therefore, EXT MNs still showed hypoglycemic effects after short-term application without drug resistance. Interestingly, the fasting blood glucose levels in all groups were approximately 15 mmol/L on Day 1 and increased to approximately 20 mmol/L from Day 2, as shown in Figure 8B. As the rats had a normal diet before Day 1, they mainly ate more at night and less during the day. In the experiment, the rats ate a lot during the day after fasting at night, resulting in the fasting blood glucose after Day 2 being approximately 5 mmol/L higher than that of Day 1.

## 4. Conclusions

The TS-MNs encapsulating EXT were successfully prepared. In this work, the gel content, drug diffusion, tip height, and implantation efficiency were evaluated by optimizing the cross-linking time, matrix solution concentration, CaCl_2_ concentration, and SA concentration. By analyzing the morphology and properties of the TS-MNs, EXT was clustered at the tips; then, the TS-MNs had sufficient mechanical strength to penetrate the skin so that the tips could be implanted into the skin for sustained release. Subsequently, in vitro release indicated that CA gel content affected drug diffusion. In addition, the release behavior of EXT from the TS-MNs conformed to the first-order kinetics. Additionally, the EXT activity was protected after MN preparation and remained above 85% after two months in the most severe 40 °C conditions. Finally, the activity of TS-MNs in vivo was evaluated. Pharmacokinetic experiments showed that the concentration of EXT could be detected within 48 h in the EXT MN group, indicating that part of the EXT was still released slowly from the gel. The hypoglycemic effect was observed to last for approximately 24 h after a single administration and remained effective after multiple administrations without drug resistance. Consequently, the TS-MNs provide tremendous convenience for patients to self-administer drugs.

## Figures and Tables

**Table 1:** Fitting of the in vitro release data.

Fitting Kinetic Model	Formulations			
	SA:EXT = 3:2		SA:EXT = 3:2	
	Equation	R^2^	Equation	R^2^
Zero-order	Q_t_ = 1.71 t + 16.27	0.7581	Q_t_ = 1.67 t + 11.96	0.8221
First-order	Q_t_ = 84.06 (1 – e^−0.08t^)	0.9878	Q_t_ = 82.40 (1 – e^−0.06t^)	0.9893
Higuchi	Q_t_ = 13.59 t^1/2^ – 1.97	0.9177	Q_t_ = 13.01 t^1/2^ – 4.86	0.9419

**Table 2:** Pharmacokinetic parameters of EXT in the groups treated with SC and EXT MNs in vivo in rats (*n* = 6).

Pharmacokinetic Parameters	EXT MN Group	SC Injection Group
T_max_ (h)	0.5	0.5
C_max_ (ng/mL)	13.60 ± 2.95	16.68 ± 4.87
AUC_0-t_ (ng·h/mL)	55.17	28.01

## Data Availability

Data is available in the text.

## Supplementary Materials

The following supporting information can be downloaded at: https://www.mdpi.com/article/10.3390/pharmaceutics14061255/s1, Figure S1: Schematic representation of experimental operation method in Section 2.3.
