# Supplementary material for: Two-Layer Sustained-Release Microneedles Encapsulating Exenatide for Type 2 Diabetes Treatment
Source: Pharmaceutics. 2022 Jun 13;14(6):1255. doi: 10.3390/pharmaceutics14061255 (PMC9230706; doi:10.3390/pharmaceutics14061255)
Supplement: Supplementary file 1 [file pharmaceutics-14-01255-s001.zip › pharmaceutics-1693310-supplementary.pdf]

pharmaceutics

MDPI

Supplementary Materials

Two-Layer Sustained-Release Microneedles Encapsulating Exenatide for Type 2 Diabetes Treatment

Han Liu, Suohui Zhang, Zequan Zhou, Mengzhen Xing and Yunhua Gao

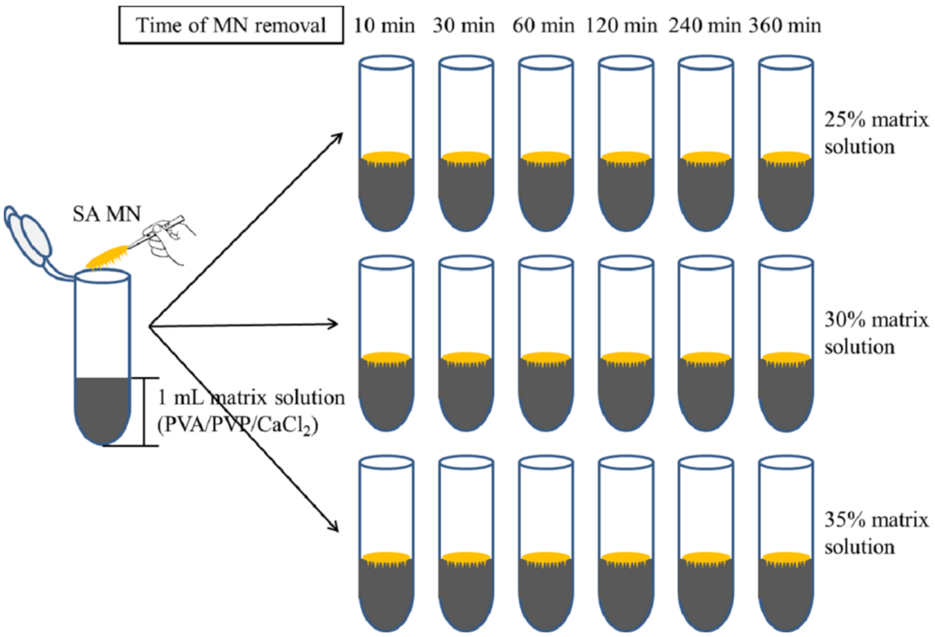

Figure S1. Schematic representation of experimental operation method in Section 2.3.
